# Experimental Characterization and Modeling Multifunctional Properties of Epoxy/Graphene Oxide Nanocomposites

**DOI:** 10.3390/polym13162831

**Published:** 2021-08-23

**Authors:** Kakur Naresh, Kamran A. Khan, Rehan Umer

**Affiliations:** Department of Aerospace Engineering, Khalifa University of Science and Technology, Abu Dhabi 127788, United Arab Emirates; naresh.kakur@ku.ac.ae (K.N.); rehan.umer@ku.ac.ae (R.U.)

**Keywords:** graphene oxide, transmission electron microscopy, mechanical properties, thermal properties, theoretical modeling

## Abstract

Thermomechanical modeling of epoxy/graphene oxide under quasi-static and dynamic loading requires thermo-mechanical properties such as Young’s modulus, Poisson’s ratio, thermal conductivity, and frequency-temperature dependent viscoelastic properties. In this study, the effects of different graphene oxide (GO) concentrations (0.05, 0.1, and 0.2 wt%) within an epoxy matrix on several mechanical and thermal properties were investigated. The distribution of GO fillers in the epoxy was investigated using transmission electron microscopy (TEM). The digital image correlation (DIC) technique was employed during the tensile testing to determine Young’s modulus and Poisson’s ratio. Analytical models were used to predict Young’s modulus and thermal conductivity, with an error of less than 13% and 9%, respectively. Frequency–temperature dependent phenomenological models were proposed to predict the storage moduli and loss tangent, with a reasonable agreement with experimental data. A relatively high storage modulus, heat-resistance index (T_HRI_), and thermal conductivity were observed in 0.2 wt% nanocomposite samples compared with pure epoxy and other lower concentration GO nanocomposites. A high *T*_HRI_ and derivative of thermogravimetric analysis peak temperatures (*T*_m1_ and *T*_m2_) were exhibited by adding nano-fillers in the epoxy, which confirms higher thermal stability of nanocomposites than that of pristine epoxy.

## 1. Introduction

Epoxy resin is one of the most commonly used thermosetting matrix systems in aircraft and automotive components, marine structures, and several coatings and adhesives due to its low shrinkage, high strength, and excellent physical and chemical stability [1]. However, owing to the low thermal conductivity of epoxies, it is difficult to dissipate heat generated when subjected to frequency-dependent loading of structures [2,3]. As a result, crack tends to form during loading, and ultimately reduces the life of the component [4,5]. The addition of suitable nano-fillers in the epoxy resin was found to enhance the thermal conductivity, temperature–frequency-dependent viscoelastic properties, fatigue life, and fracture toughness [6]. The high surface area of nano-fillers can potentially improve the interfacial bonding with the polymer chains, resulting in enhanced thermal stability and energy absorption capability of the nanocomposite [7,8,9]. Recently, nanoparticles such as carbon black, carbon nanotubes (CNTs), and graphene have found potential use as fillers in polymer matrix composites (PMCs) [10,11]. Therefore, experimental characterization and modeling of mechanical and other multifunctional properties is of great interest to many industries [12].

In particular, graphene oxide (GO) and CNTs possess higher improvement in mechanical, thermal, and impact properties among the other types of fillers [13]. However, the cost of CNTs is relatively high and, more importantly, the high viscosity of the CNTs/epoxy mixture limits adding higher amounts of CNTs into the matrix [14]. In contrast to CNTs/epoxy mixtures, the low viscosity of the epoxy/GO mixtures at similar mixing ratios facilitates a better processibility [15] even for a higher range of nano-filler addition [16]. Rafiee et al. [17] investigated and compared the mechanical properties of pristine epoxy and low weight percentage (0.1%) of GO fillers and single and multi-walled CNTs added into the epoxy matrix. It was reported that the tensile strength, modulus, and mode I fracture toughness values of GO-reinforced nanocomposites were significantly higher than those of single- and multi-walled CNTs-reinforced nanocomposites. Owing to these benefits, GO offers potential applications in the fields of aerospace, bioengineering, electrical and electronic devices, energy and battery applications, and so on [18,19,20]. Moreover, their unique mechanical characteristics combined with high surface area and large aspect ratio have great potential for next-generation polymer matrix composites [21].

Recently, several multifunctional properties of GO-based nanocomposites have been investigated. Qi et al. [22] studied the tensile, flexural, impact, and thermal properties of different weight percentages (0, 1, 3, 5, and 7 wt%) of GO mixed in the epoxy matrix to manufacture nanocomposites. It was reported that the tensile and impact properties increased as the filler content increased up to 3 wt%, and then the properties decreased with further addition of the nano-filler in the matrix. However, the flexural properties and thermal stability, which were measured based on weight reduction using a thermogravimetric analysis (TGA), increased up to 5 wt% of nano-filler content and then decreased. The decrease in properties at a higher filler content was attributed to non-uniform dispersion owing to agglomerations in the resin. Olowojoba et al. [23] studied the tensile and thermal properties of different weight percents of reduced graphene oxide (rGO) filler from 0 wt% to 0.06 wt% in the epoxy resin. As the filler content increased, an increase in tensile modulus and thermal conductivity was observed, whereas a decrease in tensile strength, fracture strain, and T_g_ were also reported. Liu et al. [24] studied the effects of different concentrations (0, 0.1, 0.5, 1, and 1.5 wt%) of GO on mechanical and thermal properties of epoxy resin. It was found that the flexural modulus increased continuously, whereas T_g_ decreased with the addition of GO from 0 wt% to 1.5 wt% in the epoxy matrix. However, the flexural strength and impact energy increased up to 1 wt% of GO addition and then decreased after further addition of GO. The drop in properties at 1.5 wt% GO was mainly owing to the agglomeration of GO in the resin at a high filler content. The addition of modified graphene nano-fillers (GO is functionalized with amino groups) of 0.98 vol% in the epoxy resin resulted in an increase in fracture toughness by 387% [25]. Moriche et al. [26] reported an increase in thermal conductivity of approximately 206% and 306% by adding 8 and 10 wt%, respectively, of GO nano fillers in the epoxy resin.

Monteserin et al. [27] studied the dynamic mechanical properties of different weight fractions (0, 0.5, 2, and 5 wt%) of GO and rGO incorporated in epoxy matrix, over a temperature range of −50 °C to 250 °C at 1 Hz frequency. A continuous decrease in [(tan δ)_max_] values and an increase in storage modulus values up to 0.5 wt% of GO content were observed, and then decreases with further addition of GO into the epoxy matrix. This confirms the drawback of incorporating higher concentrations of nano-fillers into the polymer matrix. Higher T_g_ values were also found in rGO samples compared with the GO-modified matrix, although not much variation was found in storage modulus and loss tangent. Tang et al. [28] performed dynamic mechanical experiments on different weight percentages (0, 0.05, and 0.1 wt%) of GO added in the epoxy resin. The T_g_ decreased continuously with the increase in nano-filler content in the epoxy. However, the initial storage modulus decreases up to 0.05 wt% from 0 wt%, and then increases with further increase in the nano-filler.

Yu et al. [29] investigated the mechanical and thermal behavior of GO-incorporated shape memory epoxy nanocomposites. Higher values of tensile strength, Young’s modulus, and storage modulus than pristine epoxy resin were observed, whereas lower values of fracture strain and peak loss tangent [(tan δ) _max_] at a filler content of 1.2 wt% were reported. In a recent study [30], it was reported that GO nano fillers improved the self-healing properties of the polymer matrix owing to its shape memory effect. More importantly, GO offers excellent barrier properties such as electromagnetic interference (EMI) shielding effectiveness as no other filler including CNT can offer this functionality better than GO [31]. Song et al. [32] studied multi-functional properties (thermal and electrical properties and EMI shielding performance) of pristine epoxy and different weight percentages of honeycomb structural rGO-MXene (rGMH)/epoxy nanocomposites. The storage modulus, heat-resistance index, and EMI shielding values measured in the pristine epoxy sample were ~6 GPa, 173.5 °C, and 2 dB, respectively. However, these values were increased to 7.56 GPa, 184.1 °C, and above 30 dB (depends on cell size), respectively, with the addition of rGO of 1.2 wt% and MXene of 3.3 wt% in the epoxy. Wang et al. [3] reported that thermally annealed graphene aerogels (TAGA) added in epoxy nanocomposites provide better thermal and electrical properties and EMI shielding performance than pristine epoxy. Further details about polymer-based EMI shielding composites have been reported in recent articles [33,34,35].

Despite the availability of several experimental studies related to epoxy/GO nanocomposites, a limited number of studies are available for modeling the multifunctional properties such as the static and dynamic mechanical properties and thermal conductivity of epoxy/GO nanocomposites. Some researchers have attempted modeling the mechanical properties [36,37] and thermal conductivity of epoxy/GO nanocomposites [38,39] containing relatively high weight percentages of GO fillers. High weight percentages of GO fillers cause resin embrittlement, which lead to decrease in the mechanical properties and especially a decrease in the pseudo-ductility [23].

Moreover, to the best of our knowledge, there are no studies available on modeling the temperature–frequency-dependent viscoelastic properties of epoxy/GO nanocomposites. Therefore, our primary goal is not to enhance the properties by modifying the interaction between filler and matrix; instead, this study is aimed at modeling multifunctional properties of relatively low weight percentages (up to 0.2 wt%) of GO filler incorporated in the epoxy matrix. This work is novel in aspects related to the thermo-mechanical modeling and multifunctional properties analysis of epoxy/GO nanocomposites. In this study, we experimentally investigated the tensile properties based on DIC and performed thermomechanical analysis including DMA, DSC, TGA, thermal conductivity, and EMI shielding effectiveness of epoxy/GO nanocomposites. Besides, the theoretical models were used to predict the tensile modulus, thermal conductivity, and temperature–frequency-dependent viscoelastic properties. The DIC was used to report Poisson’s ratio of nanocomposites, which is a useful property to be used as an input parameter in multiscale modeling.

## 2. Materials’ and Sample Fabrication

**Figure 1:**
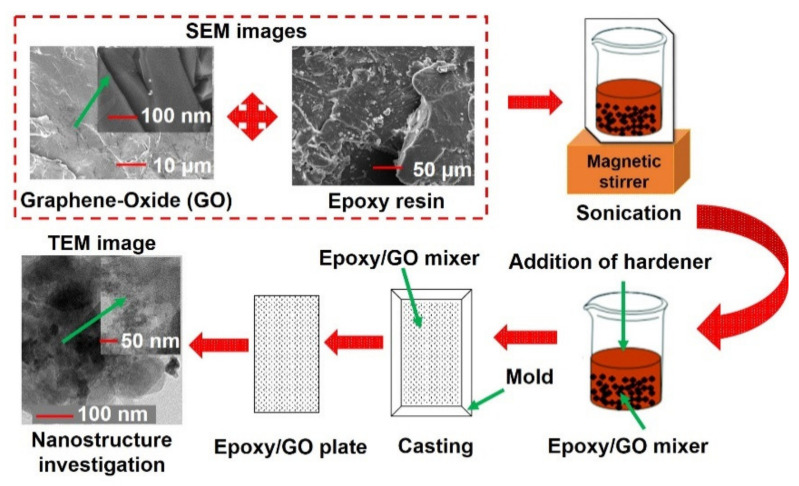
Schematic representation of the fabrication procedure used for epoxy/GO nanocomposites.

Recently, epoxy/GO nanocomposites have found interest in several fiber-reinforced polymer composite applications. The materials used in this study were graphene oxide (GO) synthesized from graphite powder (supplied from Sigma-Aldrich Co. Ltd. Abu Dhabi, U.A.E.) through the modified Hummers method. Gurit’s Prime 20LV™ epoxy resin and hardener were used as the matrix material. The procedure for GO preparation was reported in our previous study [40]. The stepwise fabrication procedure for different weight contents of GO mixed epoxy samples is given in Figure 1.

Initially, GO and epoxy resin was thoroughly mixed using a magnetic stirrer for 2 h, and then the hardener was added into the mixture. The resin to hardener ratio used was 100:28 by weight. After that, the samples were poured into a metallic casting mold and cured at room temperature for 24 h. Further, the samples were post-cured at 65 °C for 7 h before performing the experiments. The same procedure was used for preparing different weight concentrations (0, 0.05, 0.1, and 0.2%) of epoxy/GO nanocomposite samples. The manufactured samples are shown in Figure 4. Before performing the experiments, the dispersion of GO nano-fillers in the epoxy matrix was studied using a high-resolution transmission electron microscopy (Model: FEI Tecnai G^2^ F20 S-TWIN HR(S). The TEM images were captured at different magnifications, which are shown in Figure 3 in Section 5.1. A good uniform dispersion of GO in the epoxy resin was observed.

## 3. Experimental Procedures

### 3.1. Thermo-Mechanical Experiments

#### 3.1.1. Tensile Testing

**Figure 2:**
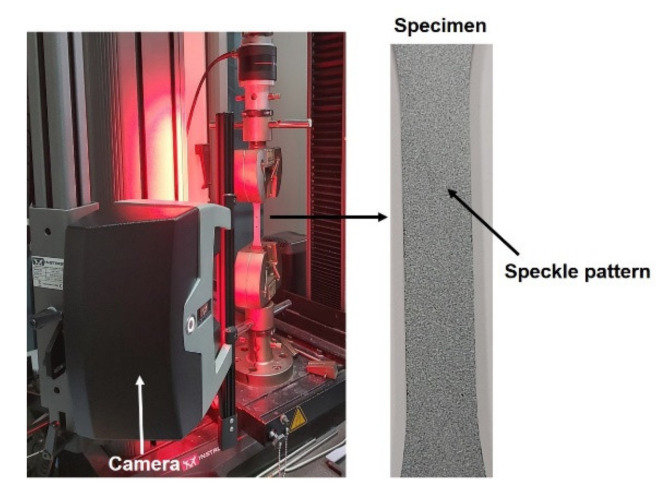
Instron UTM equipped with tensile fixture and non-contact strain measurement facility.

Tensile tests were performed as per the ASTM D638 on all manufactured samples using Instron (Model: 5969) universal testing machine (UTM). These tests were performed at the displacement rate of 2 mm/min. The displacement was measured in situ by employing the hybrid non-contact displacement measurement techniques such as two-point and full-field (DIC) using the laser-based advanced video extensometer (AVE), as shown in Figure 2.

The AVE camera equipped with Fujinon HF-35-HA-1B series lenses with the focal length ranging from 9 mm to 35 mm was used. A maximum number of 50 fps (frames per second) can be captured using this camera. A focal length of 35 mm was used in this study for capturing the images. The width and thickness of the samples in the gauge portion used were 13 mm and 3 mm, respectively. Finally, the captured images were post-processed using the DIC replay software to obtain the required displacement field and strain distribution in the region of interest. Further details about the DIC procedure employed and the advantages of non-contact strain measurement techniques over contact strain measurement techniques are given in our previous study [41,42,43].

#### 3.1.2. Frequency–Temperature-Dependent Viscoelastic Properties

The thermal tests under the cyclic load were also performed on different weight fractions of epoxy/GO nanocomposites using the dynamic mechanical analyzer (DMA; Model: PerkinElmer DMA8000) under three-point bending. The dimensions used for this test were 20 mm × 7 mm × ~3 mm (length, width, and thickness, respectively). The tests were performed at different frequencies, namely, 0.1, 1, 5, 10, 15, and 30 Hz, over the temperature range from 23 °C to 150 °C at a heating rate of 2 °C/min.

#### 3.1.3. Thermal Conductivity (TC) Measurements

The thermal conductivity experiments were performed on pure epoxy and GO modified samples using the hot disk thermal constants analyzer (Model: TPS 2500 S). The specimen dimensions used for this test were 45 mm × 45 mm (length and width). The thickness of the samples used was approximately 3 mm.

### 3.2. Thermal Characterization

#### 3.2.1. Differential Scanning Calorimetry (DSC)

The heat flow experiments were performed on different weight percentages of GO reinforced epoxy samples using the TA instrument (Model: SDT Q600). These experiments were performed over the temperature range of 23 °C to 150 °C, at a heating rate of 10 °C/min.

#### 3.2.2. Thermogravimetric Analysis (TGA)

The percentage reduction in the mass of different weight percentages of GO-reinforced epoxy samples was studied through TGA analysis using the TA instrument (Model: SDT Q600). These experiments were performed over the temperature range of 23 °C to 900 °C, at a heating rate of 10 °C/min.

### 3.3. Electromagnetic Interference (EMI) Shielding

EMI shielding effectiveness (SE) experiments were performed on pure epoxy and GO nanocomposites using a vector network analyzer (Model: Agilent, E5071CENA). These tests were performed over a frequency range of 8.40 GHz to 12.20 GHz. The dimensions of the specimen used for this test were 22.5 mm and 12 mm (length and width). The thickness of the specimen used was approximately 3 mm.

## 4. Theoretical Modeling

### 4.1. Young’s Modulus

The tensile modulus of epoxy/GO nanocomposites ($E_{\mathrm{tc}}$) with randomly oriented nano-fillers can be predicted using the Halpin–Tsai model [17,37], which is given by the following Equation (1):

(1) $$E_{\mathrm{tc}}=\frac{3}{8}\;E_{L}+\frac{5}{8}\;E_{T}$$

where $E_{L}=E_{\mathrm{tm}}\left[\;\frac{1+\eta_{L}\;\xi \;V_{p}}{1-\eta_{L}\;V_{p}}\;\right]$*;* $E_{T}=E_{\mathrm{tm}}\left[\;\frac{1+\eta_{T}\;V_{p}}{1-\eta_{T}\;V_{p}}\;\right]$*;* $\eta_{L}=(\;\frac{\frac{E_{p}}{E_{\mathrm{tm}}}-1}{\frac{E_{p}}{E_{\mathrm{tm}}}+\xi }\;$*);* $\eta_{T}=(\;\frac{\frac{E_{p}}{E_{\mathrm{tm}}}-1}{\frac{E_{p}}{E_{\mathrm{tm}}}+2}\;$*)*; $E_{\mathrm{tm}}$ and $E_{p}$ are the tensile moduli of pristine epoxy and GO nano-filler, respectively. The tensile modulus of pristine epoxy is 2.73 GPa, which was measured in this study using the experiments. The tensile modulus of GO nano filler is approximately 250 GPa [21]. $V_{p}$ is the volume fraction of GO filler. The densities of epoxy and GO filler are 1350 kg/m^3^ and 2250 kg/m^3^, respectively [36]. $\xi =\frac{2}{3}\;\frac{l_{p}}{t_{p}}$; l_p_ and t_p_ are the length and thickness of the GO filler, respectively. The values of l_p_ and t_p_ are approximately 0.85 μm and 2.696 nm, respectively [37].

### 4.2. Frequency–Temperature-Dependent Viscoelastic Modeling

The viscoelastic properties such as storage modulus $\left[E\prime \left(T,\;f\right)\right]$, loss modulus $\left[E\prime \prime \left(T,\;f\right)\right]$, and damping term (tan δ) called the loss factor are functions of both the temperature and frequency. When the thermal load is applied to polymer or polymer composite samples cyclically, those materials restore some amount of stiffness owing to their elasticity, while losing some quantity of stiffness owing to damping [42]. The elastic behavior of the viscoelastic material during cyclic loading can be indicated as the storage modulus. Maximum storage modulus exists at room temperature, which is called the initial storage modulus $E_{\max}^{\prime }\left(T,\;f\right)$. The stiffness loss during sinusoidal thermal load can be represented as loss or viscous modulus. The loss factor is the other essential viscoelastic parameter that can be obtained from the ratio of$\;E\prime \prime \left(T,\;f\right)$ and $E\prime \left(T,\;f\right)$ [44]. The procedure for predicting these viscoelastic properties is discussed in the sub-sections.

#### 4.2.1. Effect of Frequency on Storage Modulus and Glass Transition Temperature 

In this section, the theoretical models for predicting the effect of frequency on storage modulus and glass transition temperature (T_g_) of pristine epoxy and epoxy/GO nanocomposites are discussed. The effect of frequency (f) on storage modulus can be determined using Equation (2), which is given by [45]

(2) $$E\prime \;\left(f\right)={E^{\prime }}^{\mathrm{ref}}\;\left(f\right)\;\left(1+\zeta \;log\;(\frac{f}{f^{\mathrm{ref}}}\right))\;\;$$

where $E\prime^{\mathrm{ref}}\;$is the instantaneous storage modulus at the reference frequency ($f^{\mathrm{ref}})$ of 1 Hz. It is experimentally measured using the DMA. ζ is the frequency sensitivity coefficient.

The glass transition temperature of polymers and their nanocomposites can be classified as *T*_g_ (E′) _middle_ or *T*_mg_ corresponding to the inflection point of storage modulus, *T*_g_ (*E*″)_max_ corresponding to the peak value of *E*″, and *T*_g_ (tan δ)_max_ corresponding to peak values of tan δ. *T*_g_ (tan δ)_max_ is the highest one and *T*_g_ (E′)_middle_ is the lowest one among these three glass transition temperature values. Most researchers reported *T*_g_ as the temperature obtained from loss modulus and loss factor peak values [42]. The shift in *T*_g_ values with the increase in frequency can be studied using the Arrhenius model via the activation energy expression and is given by [46]

(3) $$ln\;\left(f\right)=-\frac{1}{R}\left(\frac{E_{a}}{\;T_{g}}\right)+ln\;\left(N\right)\;$$

where $f,\;E_{a}$, R, and *N* are the applied frequency, activation energy, gas constant, and pre-exponential factor, respectively. Th strain line equation is (y = mx + c). y = ln (f) and x = $\left(\frac{1}{T_{g}}\right)$; m is the slope = −$\left(\frac{E_{a}}{R}\right)$; the activation energy can be calculated in terms of the slope of $\ln\;\left(f\right)\;\mathrm{vs}.\;1/\left(T_{g}\right)$ and is given by [47]

(4) $$E_{a}=-R\times slope\;\;$$

#### 4.2.2. Effect of Volume Fraction on the Storage Modulus

In this section, the theoretical models for predicting the effect of volume fraction on the storage modulus of epoxy/GO nanocomposites are discussed. The storage modulus of nanocomposites can be predicted using Einstein’s model [48,49], which can be given by

(5) $$E^{\prime }=E_{m\;}^{1}\left(1+V_{p}\right)\;$$

where $E_{m\;}^{1}$ and $V_{p}$ are the matrix storage modulus and volume fraction of nano-fillers, respectively. Guth [50] modified Equation (5) to reduce the deviation between the predicted and experimental storage modulus values, which is given by

(6) $$E^{\prime }=E_{m\;}^{1}\left(1+2.5\;V_{p}+14.1\;V_{p}^{2}\;\right)\;$$

However, in these models, the volume fraction of nanoparticles is the only controlling parameter. However, it is also important to use theoretical models that consider the temperature and frequency effects, as the viscoelastic properties of polymers and their nanocomposites rely on these parameters.

#### 4.2.3. Effects of Temperature and Frequency on Storage and Loss Moduli

In this section, the theoretical models for predicting the effects of temperature and frequency on storage and loss moduli of pristine epoxy and epoxy/GO nanocomposites are discussed. The two-parameter viscoelastic model for polymers and polymer matrix composites is proposed by Feng et al. [51,52] to predict the complete history of storage and loss modulus curves, which are given by

(7) $$E\prime \left(T,\;f\right)=E_{G}^{\prime }-\frac{E_{G}^{\prime }-E_{R}^{\prime }}{{\left[\left(2^{q}-1\right)\;e^{p\;q\;\left(T_{m}{}_{g}-T\right)}+1\right]}^{\left(\frac{1}{q}\right)}\;}\;$$

(8) $$E\prime \prime \left(T,\;f\right)=C\;\frac{p\;\left(2^{q}-1\right)\;e^{p\;q\;\left(T_{m}{}_{g}-T\right)}}{{\left[\left(2^{q}-1\right)\;e^{p\;q\;\left(T_{m}{}_{g}-T\right)}+1\right]}^{\left(\frac{1}{q}+1\right)}\;}\;$$

where $C=\frac{\left(\;E\prime \prime \right)\max}{{\left[\frac{p\;q}{q+1}\right]}^{\left(\frac{1}{q}+1\right)}\;}$; $E_{G}^{\prime }$ is the glassy storage modulus and $E_{R}^{\prime }$ is the rubbery storage modulus; and *T* is the temperature. p and *q* are the intrinsic growth rate and the symmetry of the glass transition region, respectively. These parameters are determined by curve fitting degree of glass transition $\left(\varphi \right)$ versus T using Equation (9) [53]:

(9) $$\left(\varphi \right)=\frac{1}{{\left[\left(2^{q}-1\right)e^{p\;q\;\left(T_{m}{}_{g}-T\right)}+1\right]}^{\left(\frac{1}{q}\right)}\;}\;$$

### 4.3. Thermal Conductivity Modeling

The thermal conductivity of epoxy/GO nanocomposites can be determined through the Russell model using Equation (10), which is given by [38]

(10) $$\lambda_{c}=\lambda_{m}\left[\;\frac{V_{p}{}^{2/3}+\frac{\lambda_{m}}{\lambda_{p\;\left(1-V_{p}{}^{2/3}\;\right)}}}{V_{p}{}^{2/3}-V_{p}+\frac{\lambda_{m}}{\lambda_{p\;\left(1-V_{p}{}^{2/3}\;\right)}}}\;\right]\;$$

where $\lambda_{c},\;\;\lambda_{m}$, and $\lambda_{p}$ are the thermal conductivity of composites, pristine epoxy, and GO filler, respectively. $\;\lambda_{m}$ = 0.258 W/mK is measured in this study through the experiments. $\lambda_{p}$ = ~18 W/mK is taken from the literature [54].

### 4.4. EMI Shielding Modeling

In recent times, the EMI shielding effectiveness using GO films embedded in the polymer matrix composites has been investigated [55]. Here, we conducted the EMI shielding effectiveness measurements on pure epoxy and GO nanocomposites. The total EMI shielding (SE_T_) is the summation of EMI shield effectiveness of absorption (*SE_A_*) and reflection (*SE_R_*) phases [56]. The higher the total EMI shielding values, the lower the possible penetration of the radio or microwave radiation in the material [33,57]. These parameters (*SE_A_* and *SE_R_*) can be calculated using the scattering or S- parameters: *S*_11_, *S*_12_, *S*_21_, and *S*_22_. The first and second numbers in the subscript of S-parameters refer to the responding and incident ports, respectively. The EMI shielding effectiveness values of absorption and reflection can be calculated using Equations (11) and (12), respectively [58]:

(11) $$SE_{A}=10\;Log_{10}\;\left(\frac{1-{\left|S_{11}\right|}^{2}}{{\left|S_{12}\right|}^{2}}\right)\;$$

(12) $$SE_{R}=10\;Log_{10}\;\left(\frac{1}{1-{\left|S_{11}\right|}^{2}}\right)\;$$

## 5. Results and Discussions

### 5.1. Transmission Electron Microscopy (TEM) Studies

**Figure 3:**
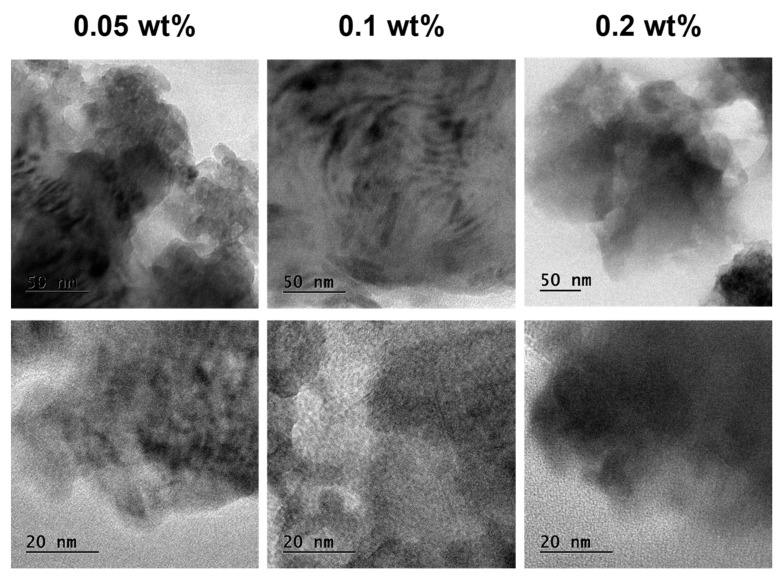
Transmission electron microscopy images of different nanocomposites.

Figure 3 shows the transmission electron microscopy (TEM) images of different weight percentages of GO filler-reinforced epoxy samples. TEM images show the exfoliated nanostructure in the epoxy resin. However, relatively, the samples of higher magnifications (20 nm) show more uniform dispersion of GO fillers in the epoxy compared with samples of lower magnifications (50 nm). In particular, GO fillers in samples of lower weight percentages such as 0.05 and 0.1 wt% are more uniformly distributed compared with that in 0.2 wt%. This is because of low agglomerations that took place in the epoxy at low filler loadings of 0.05 and 0.1 wt% than at 0.2 wt%.

### 5.2. Tensile Test Results

**Figure 4:**
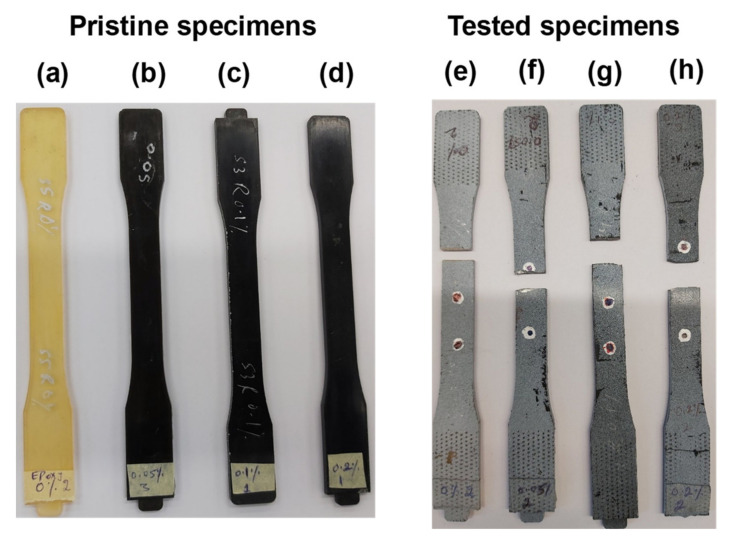
Typical different weight percentages of GO-reinforced epoxy specimens: 0% (**a**,**e**), 0.05% (**b**,**f**), 0.1% (**c**,**g**), and 0.2% (**d**,**h**).

In this section, the tensile properties and the Poisson’s ratio of pristine epoxy (0 wt%) and epoxy/GO nanocomposites (0.05, 0.1 and 0.2 wt%) are presented and discussed. Figure 4a–d shows the Pristine specimens of epoxy and different nanocomposites. Figure 4e–h shows the tensile tested specimens of epoxy and different nanocomposites. It is observed from the tested specimens that the failure occurred in the gauge portion.

**Figure 5:**
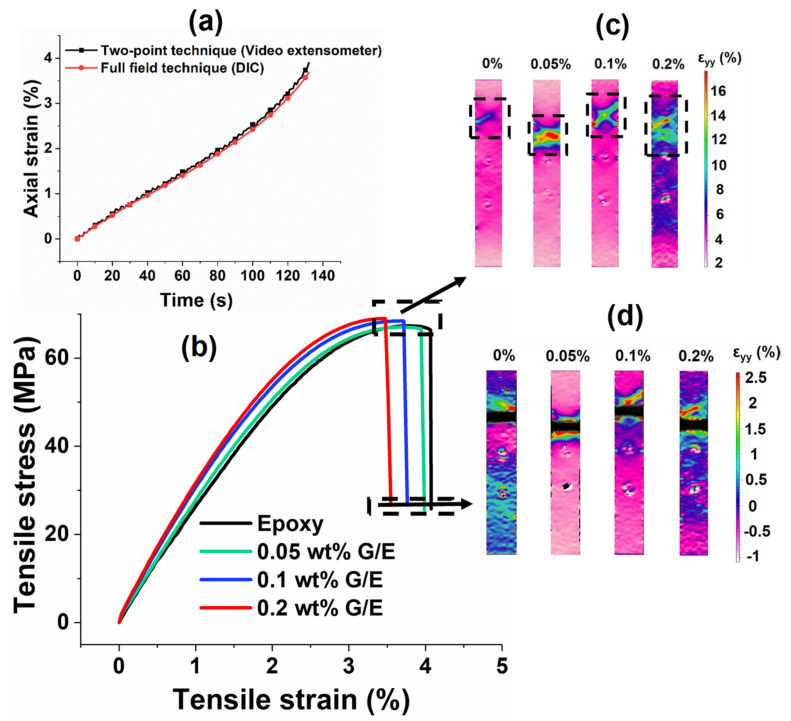
(**a**) Comparison of two-point and full-field axial strain measurement techniques, (**b**) tensile stress vs. strain, (**c**) axial strain (ε_yy_) contour plots of all samples at maximum stress, and (**d**) fracture surfaces shown in black color at the end of the test.

Figure 5a shows the axial strain of a 0.1 wt% GO sample measured using different strain measurement techniques. An excellent correlation of the axial strain values measured in both a two-point technique with the help of a video extensometer and a full-field technique using DIC is observed. However, the smoothness of the axial strain curve measured using DIC is higher in comparison with the two-point technique. Therefore, the DIC results are used for determining the tensile modulus of pristine epoxy and epoxy/GO nanocomposites. Figure 5b presents the tensile stress versus strain curves of the pristine epoxy and epoxy/GO nanocomposites. Figure 5c,d shows the strain contours obtained using the DIC technique at different loading conditions such as the maximum load at failure.

It can be observed from Figure 5b that the tensile strength and modulus increase, whereas the percentage failure strain decreases with an increase in GO content from 0 wt% to 0.2 wt%. Similar trends were observed by Yu et al. [29] for tensile strength and modulus; in their study, a further decrease in failure strain was observed at GO content up to 0.8 wt%. The strain contour plots at maximum loads are depicted in Figure 5c, which shows that the region of crack propagation and failure increases with the increase in GO content. These results show that the brittleness in tensile samples increased as the GO content increased. The fractured surfaces of all the samples are shown in Figure 4e–h.

**Figure 6:**
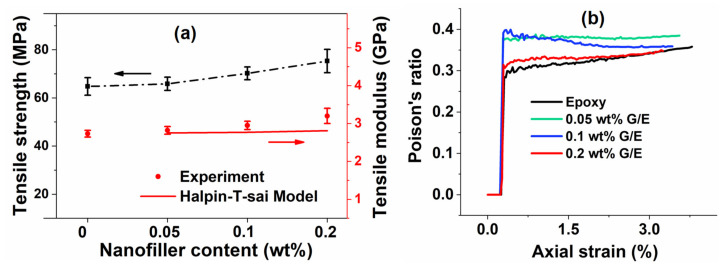
(**a**) Tensile properties at different GO contents and (**b**) Poisson’s ratio values of different nanocomposites.

Figure 6a presents the variation in tensile strength and modulus as the GO content increased from 0 wt% to 0.2 wt%. It can be observed from the figure that the average tensile strength increases by 1.7, 8.4, and 16.3% for 0.05, 0.1, and 0.2 wt% GO samples, respectively. Similar trends were reported elsewhere [59,60]. The average tensile modulus increased by 3.3, 8.1, and 17.2% for 0.05, 0.1, and 0.2 wt% GO samples, respectively. The predicted tensile modulus values of nanocomposites are given in Figure 6a, which were determined using the Halpin–Tsai model, as described by Equation (1). The percentage deviation between the experimental and predicted tensile moduli of 0.05, 0.1, and 0.2 wt% GO samples was 2.5, 6.5, and 13.8%, respectively. These results show a reasonably good agreement between the experimental and predicted tensile modulus values. Figure 6b shows the Poisson’s ratio of pristine epoxy and GO samples. The Poisson’s ratio values in the linear elastic region were found to be 0.30 in the pristine epoxy and 0.37, 0.38, and 0.32, respectively, for the GO samples. The Poisson’s ratio increased by 23.3, 26.7, and 6.7%, respectively, as the GO content increased. Similar trends were observed by Shi et al. [61] as well.

### 5.3. Frequency–Temperature-Dependent Viscoelastic Properties

The dynamic mechanical properties of pristine epoxy and GO samples for a temperature range of 23 °C to 150 °C at different frequencies (0.1, 1, 5, 10, 15, and 30 Hz) are presented here. The storage modulus and loss modulus are predicted for the corresponding temperature and frequency ranges using Equations (7) and (8), respectively. The tan δ values were determined from the predicted storage and loss moduli curves. The unknown parameters *p* and *q* in Equations (7) and (8) were obtained using a curve fitting parameter for the φ versus *T* graph using Equation (9) in MATLAB. As can be seen in Table S1 in the Supporting Document, the parameters *p* and *q* decrease with the increase in frequency from 0.1 Hz to 30 Hz. Similar trends were reported in studies in the literature [51,52,53].

#### 5.3.1. Storage and Loss Moduli

**Figure 7:**
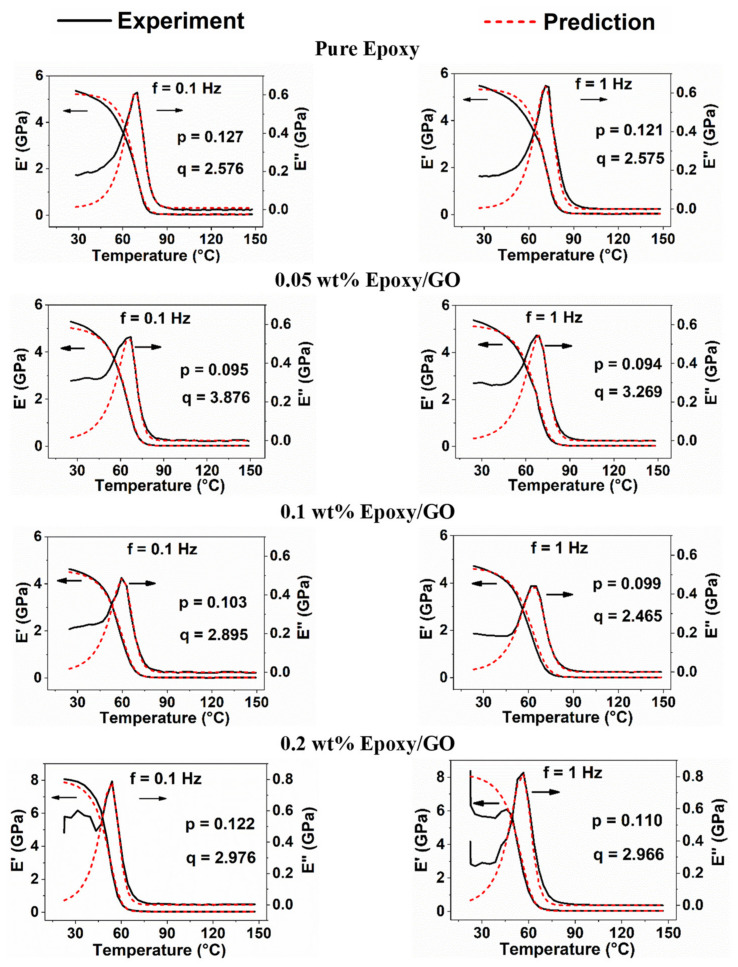
Comparison between experimental and predicted storage and loss moduli of pristine epoxy and GO samples at different frequencies.

The evolution of storage and loss moduli of pristine epoxy and different GO samples is discussed in this section. The results were obtained for a temperature of 23 °C to 150 °C at different frequencies (0.1, 1, 5, 10, 15, and 30 Hz). The results of storage and loss moduli values for all frequencies are given in Table S1 in the Supporting Document. As an example, Figure 7 shows the predicted results of the storage and loss moduli for the frequencies of 0.1 and 1 Hz. A good agreement was observed; however, the model was unable to predict the initial region of loss modulus at almost all frequencies.

Initially, both the storage and loss moduli curves are slightly horizontal at room temperature, which is mainly because of the immobility of molecules in the polymer matrix. Room temperature (~23 °C) is not sufficient to cause adverse effects such as translational and rotational movements of molecular chains in the resin. Thus, as the temperature increases, adverse effects occur and gradually increase the molecular movements in the resin. A sudden drop in E′ value is seen in Figure 7 in particular, and the degree of modulus drop is higher when the temperature exceeds T_g_ (E″). This can be attributed to material softening at higher temperatures, as the mobility of molecules changes the state from immobile at room temperature to mobile.

In contrast to the E′ curve, the E″ curve increases up to T_g_. The enhanced molecular transport phenomena in the resin above T_g_ increases the rate of mobilization [53]. Therefore, the storage and loss moduli curves decrease continuously until the end of the transition region, which can be observed in Figure 7. After that, the shape of the curve plateaus with a further increase in temperature, which can be seen throughout the rubbery region as the molecules lose their energy at this stage.

It can be observed from Table S1 that the experimental (E′)_max_ for epoxy increases from 5.3 GPa at 0.1 Hz to 5.6 GPa at 30 Hz (4.6% increase), whereas the predicted (E′)_max_ increases from 5.2 GPa at 1 Hz to 5.5 GPa at 30 Hz (5% increase). Similarly, for the same range of frequency variation, the (T_g_)_experimental_ increases from 69.7 °C to 72.8 °C (4.5% increase) and (T_g_)_predicted_ increases from 67.4 °C to 72.8 °C (8.1% increase). The experimental and predicted T_g_ values were noted from the respective peak values of the loss modulus curves. These observations confirm good correlation between experimental and predicted results. The time available for molecules to relax and rearrange within the epoxy matrix is less at high frequencies compared with low frequencies [51,53,62,63]. As a result, the storage modulus and T_g_ values are higher at the frequency of 30 Hz as compared with at 0.1 Hz. Similar trends in storage modulus and T_g_ values with the increase in frequency were also observed for 0.05 wt%, 0.1 wt%, and 0.2 wt% GO samples, as shown in Table S1.

**Figure 8:**
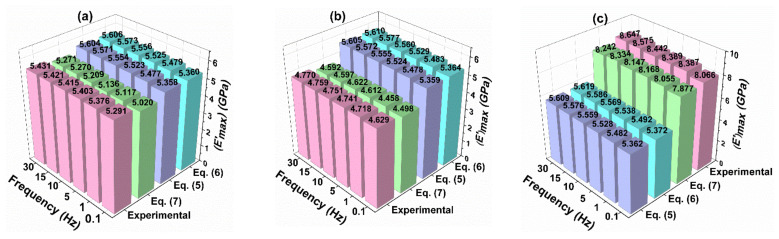
Comparison of experimental and predicted initial storage modulus values at different weight percentages of nanocomposites: (**a**) 0.05 wt%, (**b**) 0.1 wt%, and (**c**) 0.2 wt% GO.

Figure 8a–c shows the comparison of experimental and predicted initial storage modulus values of different nanocomposites. It is clear from the figure that the storage modulus values were higher for 0.2 wt% samples compared with other GO samples. An increase in storage modulus with an increase in frequency from 0.1 Hz to 30 Hz was observed for both experimental and different modeling approaches. However, the deviation of predicted values from the experiments is slightly higher for Einstein’s (Equation (5)) and modified-Einstein’s model (Equation (6)) compared with Equation (7). This is the first study that used Equation (7) for the nanocomposites, although Feng et al. [51,52] developed Equation (7) and used it for polymers and fiber-reinforced polymer composites. Hence, it can be concluded from the results (Figure 7 and Figure 8) that this model can be used for epoxy/GO nanocomposites.

#### 5.3.2. Effect of Frequency on Storage Modulus (E′)

**Figure 9:**
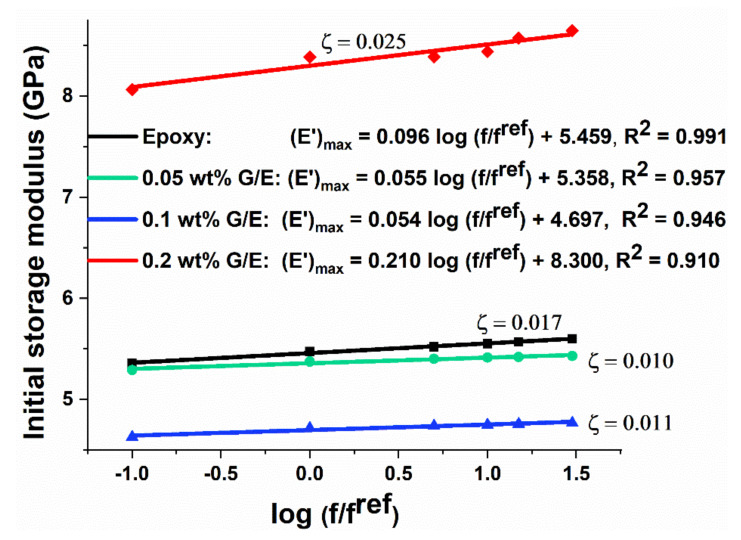
Effect of frequency on initial storage modulus of pristine epoxy and GO samples.

The coefficient of frequency sensitivity (ζ) is obtained using Equation (2), which is a linear regression equation for estimating the effect of frequency on initial storage modulus of the pristine epoxy and different GO samples, as shown in Figure 9. The higher the value of ζ, the higher the increase in initial storage modulus value with increasing frequency, and vice versa. The increment in storage modulus with the increase in frequency is large for 0.2 wt% GO samples compared with pristine epoxy and other compositions, indicated by the higher value of ζ, which brings a favorable effect.

#### 5.3.3. Experimental and Predicted T_g_ (tan δ)_max_

**Figure 10:**
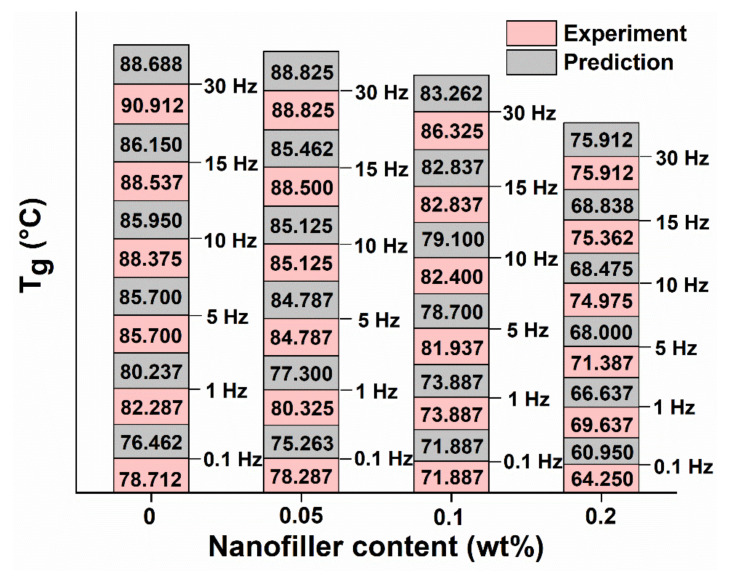
Comparison of T_g_ values between experiments and predictions for different GO content samples.

The loss tangent T_g_ (tan δ)_max_ values are shown in Figure 10. These values were obtained using Equations (7) and (8). The decrease in T_g_ values with the increase in GO content shows an improvement in self-healing properties owing to the shape memory effect of GO nano-fillers [28]. Similar trends were found elsewhere [23,24,28]. The increase in T_g_ values with the increase in frequency is also observed in both experimental and predicted values, as shown in Figure 10 and in other studies [53,64].

It can be observed from Figure 10 that the T_g_ value corresponding to experimental (tan δ) _max_ increases from 78.7 °C at 0.1 Hz to 90.9 °C at 30 Hz for pristine epoxy (15.5% increase). Similarly, the predicted T_g_ value corresponding to predicted (tan δ) _max_ increases from 76.5 °C at 0.1 Hz to 88.7 °C at 30 Hz for pristine epoxy (16% increase), which confirms the excellent correlation between both approaches.

**Figure 11:**
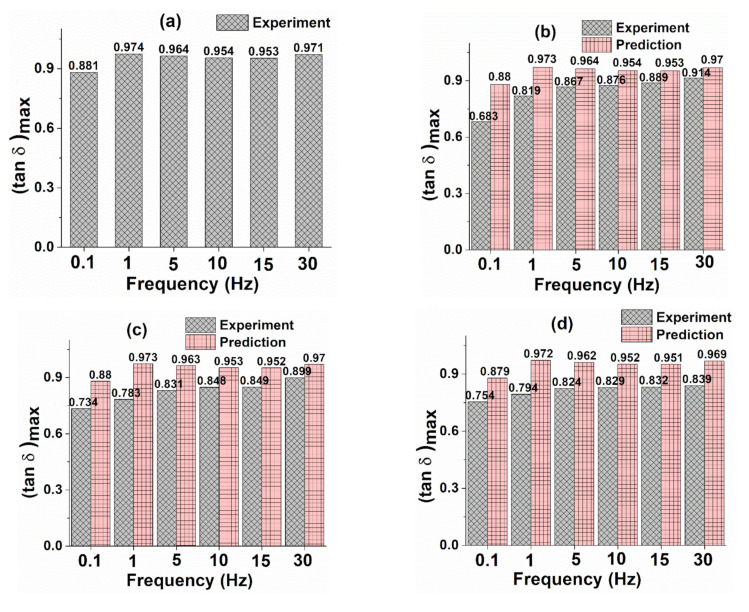
Loss factor of (**a**) pristine epoxy, (**b**) 0.05 wt%, (**c**) 0.1 wt%, and (**d**) 0.2 wt% GO samples.

Figure 11b–d shows experimental and predicted loss factor peak values of different weight percentages of GO samples for different frequencies. For determining the predicted loss factor peak values of GO samples, the filler volume fraction and loss factor values of epoxy matrix, as shown in Figure 11a, are used in Equation (13) [49]:

(13) $$tan\delta_{c}=tan\delta_{m}\;\left(1-V_{p}\right)\;$$

where, $tan\delta_{c}$ and $tan\delta_{m}$ are the loss factors of GO samples and pristine epoxy, respectively.

It can be observed from the figure that the pristine epoxy exhibits higher (tan δ) _max_ values for all frequencies compared with GO samples. This is because of an increase in interfacial bonding with the addition of GO content within the epoxy matrix [60].

#### 5.3.4. Effect of Frequency on T_g_ (tan δ)_max_

**Figure 12:**
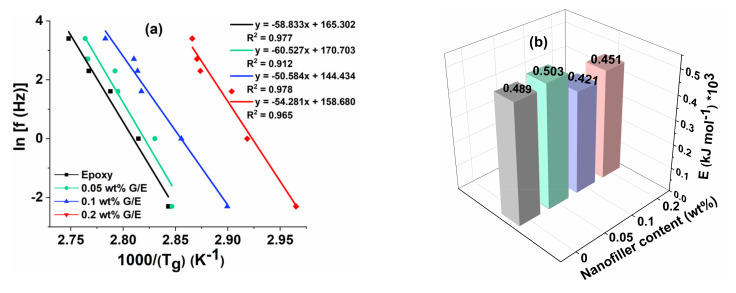
(**a**) Regression lines and (**b**) activation energy for all tested samples.

Figure 12a,b shows the variation in T_g_ (tan δ) _max_ values as the frequency (f) changes from 0.1 Hz to 30 Hz and the activation energy for all samples. These values are plotted using the Arrhenius model using Equation (3). The activation energy depends on the variation in T_g_ values as the frequency increases. The higher the change in T_g_ with the increase in frequency, the higher the resultant activation energy. It is worth noting from these figures that the change in T_g_ values are higher and almost the same in pristine epoxy and 0.05 wt% GO samples. As a result, the activation energy values were found to be higher in pristine epoxy and 0.05 wt% GO samples compared with other nanocomposites. The excellent agreement was assessed between the experimental values and curve fit from higher values (~>0.9) of the coefficient of determination (R^2^).

### 5.4. Comparison of T_g_ Using DSC and DMA

**Figure 13:**
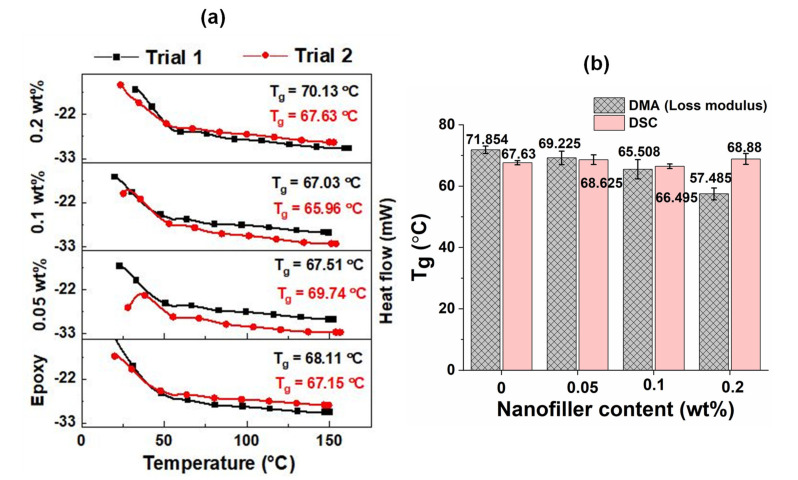
(**a**) DSC curves for pristine epoxy and different GO samples and (**b**) comparison of T_g_ values measured using DMA and DSC.

Figure 13a presents the heat flow versus temperature curves of pristine epoxy and GO nanocomposite samples, measured using the DSC. From the figure, the higher T_g_ values are seen in the pristine epoxy compared with GO samples. These measurements follow the trend of T_g_ values measured using the DMA. Figure 13b shows the comparison of T_g_ values measured through the DMA and DSC. Even though the average T_g_ values corresponding to loss modulus peak temperature and DSC correlate well at a lower amount of GO nanocomposites, higher deviation is obtained at nanocomposites of 0.2 wt% GO. However, the average T_g_ values corresponding to the loss tangent peak temperature value (71.9 °C) reasonably correlate with the T_g_ value of 68.8 °C measured using the DSC. The smaller deviation between the T_g_ values of DMA and DSC is due to the difference in heating rates. The heating rate used for DMA was 2 °C/min, whereas a heating rate of 10 °C/min was used for the DSC studies.

### 5.5. Thermogravimetric Analysis

**Figure 14:**
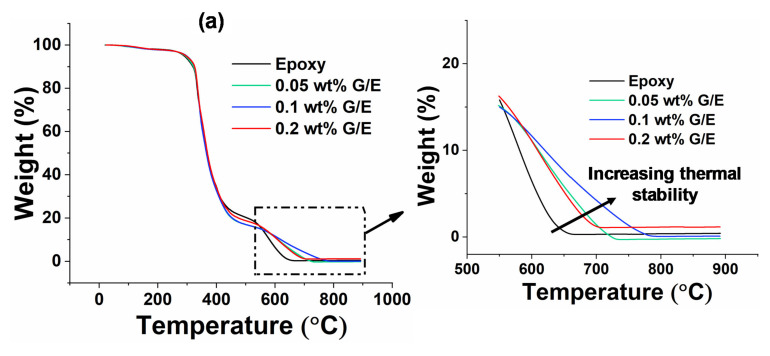

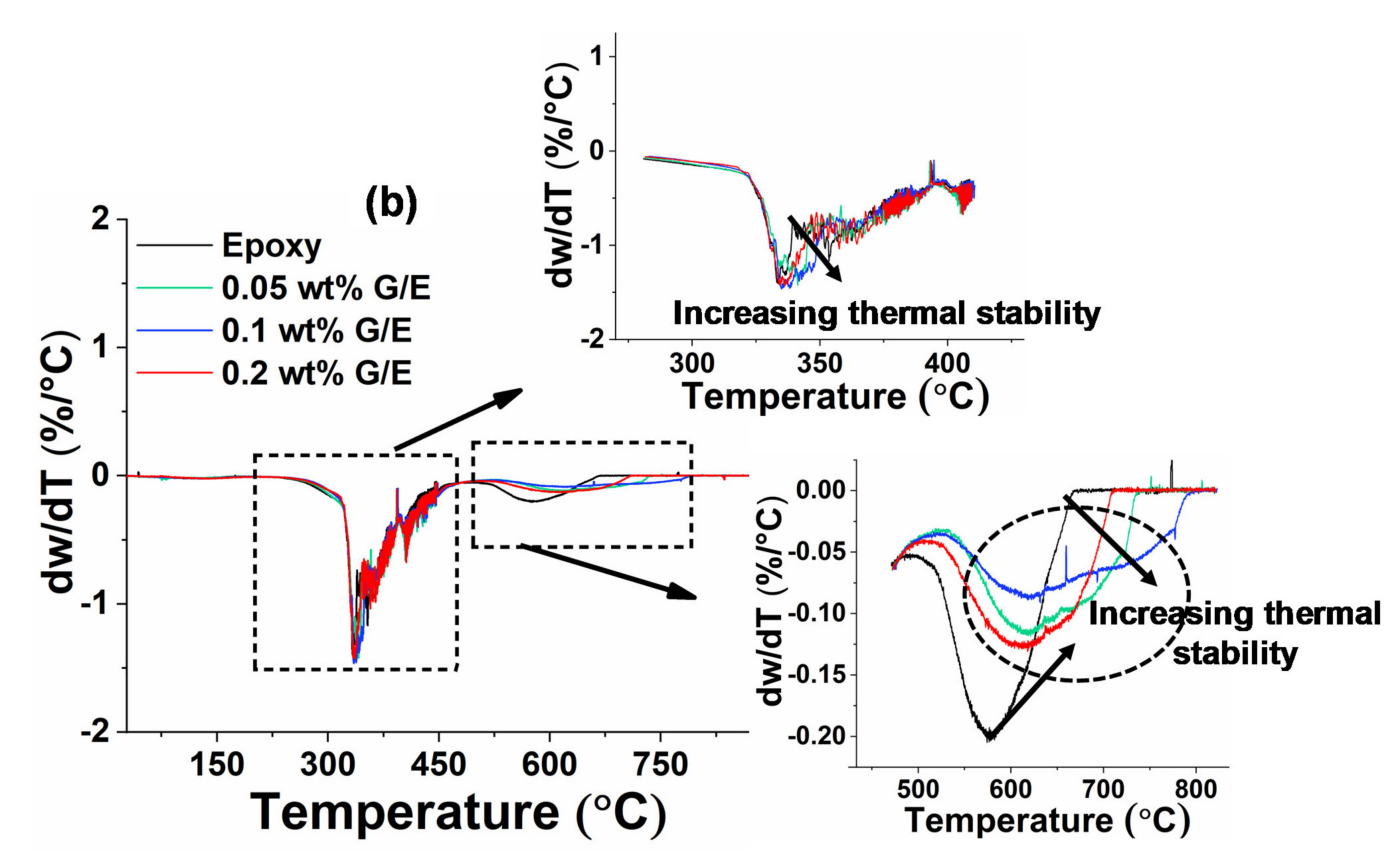
(**a**) TGA curves with zoomed section and (**b**) derivative of TGA with zoomed areas of interest.

Figure 14a shows the percentage weight loss of pristine epoxy and GO samples with the increase of temperature from 23 °C to 900 °C. Even though the percentage of weight loss is almost the same in all samples, most of the weight loss occurs earlier within the epoxy matrix at approximately from 300 °C to 620 °C. This is because of the degradation of the epoxy matrix. The close-up of Figure 14a shows that the weight loss is fast in pristine epoxy compared with GO nanocomposites, as expected. 

Figure 14b shows the derivative curve of Figure 14a, in which dw/dT indicates the change in weight of the sample with respect to temperature. The drop in weight is higher and rapid after reaching 250 °C as most of the resin decomposition takes place, which can be seen from the first peak. However, the second peak indicates the region of remaining resin decomposition. The delay in weight reduction at higher temperatures with the increase in filler content can be seen in close-up graphs of Figure 14b. Moreover, the shift in peak values (T_m1_ and T_m2_) with the increase in filler content in the epoxy can be seen clearly in the high-temperature region derivative TGA curve. These peak values (T_m1_ and T_m2_) for pristine epoxy and different nanocomposites are given in Table 1. Moreover, the shape change of the peaks from sharp to round can also be observed. These findings indicate the increase in thermal stability with the addition of GO filler in the epoxy resin. The better barrier properties of GO fillers delay the volatilization of epoxy decomposition products [65]. As a result, the molecular chain movements in the epoxy are restricted by the cross-linked structure and GO surface area, which could be the reason for increased thermal stability with the addition of GO filler [66]. The variation in residual weight and thermal stability among the nanocomposites is due to GO dispersion and interfacial interaction with the epoxy matrix [67,68]. It was reported in previous studies by several researchers that higher values of heat-resistance index and derivative of TGA peak temperatures (T_m1_ and T_m2_) are obtained by adding nanofillers in the epoxy; as a result, the thermal stability of nanocomposites was higher than the pristine epoxy [35,69]. The heat-resistance index (*T_HRI_*) is calculated using Equation (14) [3,32].

(14) $$T_{HRI}=0.49\times \left[T_{5}+0.6\times \left(T_{30}-T_{5}\right)\right]\;$$

where *T*_5_ and *T*_30_ correspond to the decomposition temperature of 5% and 30% weight loss, respectively, which are determined from Figure 14a. Table 1 shows the TGA characteristic data of pristine epoxy and different weight percentages of nanocomposites. 

From Table 1, it is observed that the values of heat-resistance index and derivative of TGA peak temperatures (*T*_m1_ and *T*_m2_) of GO fillers added in epoxy samples are higher than the pristine epoxy sample. These observations lead to the important conclusion that the thermal stability increases by adding the GO fillers in the epoxy.

### 5.6. Thermal Conductivity

**Figure 15:**
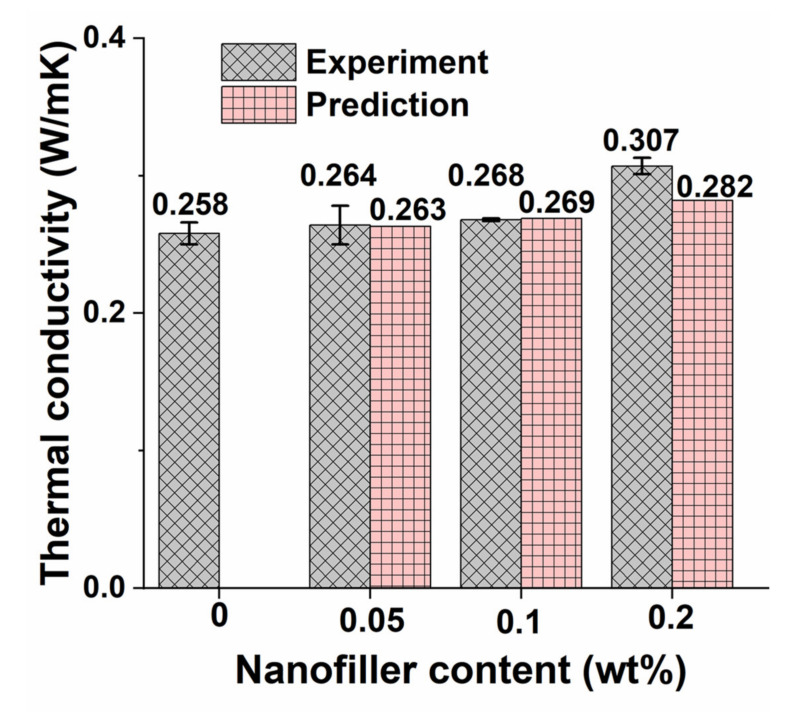
Thermal conductivity values of pristine epoxy and different GO nanocomposite samples.

Figure 15 shows the thermal conductivity values of pristine epoxy and different GO nanocomposite samples. The predicted thermal conductivity values of GO samples are determined using Equation (10). The lower thermal conductivity of pristine epoxy is due to its amorphous state [70]. It is worth observing from the figure that the average thermal conductivity increases by 2.3, 3.9, and 19% with the increase in GO content by 0.05, 0.1, 0.2 wt%, respectively. The deviation between the predicted values from the experimental values is less than 9%. The increase in thermal conductivity with the increase in filler content is due to the presence of phonons in the GO [71]. Even though GO contains both electrons and phonons, the contribution of electrons for enhancing the thermal conductivity is rarely possible [72].

### 5.7. EMI Shielding Effectiveness Measurement

**Figure 16:**
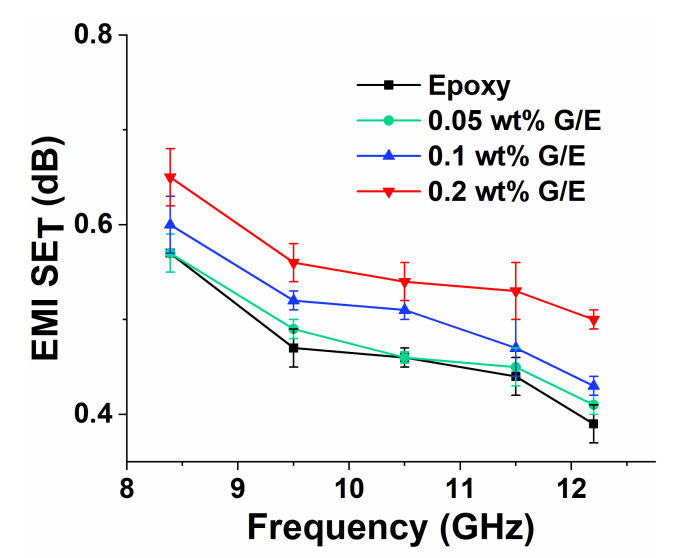
Total EMI shielding effectiveness values of pristine epoxy and different GO nanocomposites.

Figure 16 shows the comparisons of total EMI shielding effectiveness values of different GO nanocomposite samples. The EMI shielding increases by 0.6, 5.3, and 14% at 8.39 GHz with the increase in GO content of 0.05, 0.1, and 0.2 wt%, respectively. Even though an increase in total EMI shielding effectiveness values was observed with the increase in GO filler content, the magnitude of the increase is not very significant (less than 1 dB). This is because of the lower concentration of GO filler used in this study. Therefore, the concentration of GO used in this study is not sufficient to serve as an EMI shielding barrier material. Moreover, further studies are related to cross-linking coating of nanofillers [73] or adding hybrid fillers, for example, Mxene-rGO [32] and thermally annealed MXene aerogels (TCTA) [74] and thermally annealed graphene aerogels [33], in the epoxy, which improve the EMI shielding performance. 

## 6. Conclusions

The present study aimed to investigate the effects of low concentrations of GO filler in the epoxy matrix on mechanical and thermal properties. Moreover, the theoretical models were used for determining the tensile modulus, Poisson’s ration, temperature–frequency-dependent viscoelastic properties, and thermal conductivity of different GO nanocomposites. The results are summarized below.

The increase in tensile strength and modulus and decrease in failure strain (%) were observed with the increase in GO nanofiller. The deviation between the predicted and experimental tensile modulus values was less than 13%. Moreover, the Poisson’s ratio increased by 23.3, 26.7, and 6.7% with the increase in GO content.

The dynamic mechanical analysis showed an increase in storage modulus and T_g_ values in all samples with the increase in frequency from 0.1 Hz to 30 Hz. A good correlation was obtained between the predicted values and experimental results. A good agreement was observed between the T_g_ values measured in all samples using the DMA and DSC.

The increase in heat-resistance index and thermal conductivity values from 157.8 °C and 0.258 W/mK to 159.4 °C and 0.307 W/mK, respectively, was observed with the increase in GO content from 0 wt% to 0.2 wt% in the epoxy. The deviation between the predicted thermal conductivity values from the experimental results is lower than 9%.

Modeling epoxy/GO nanocomposites using the finite element method (FEM)-based approach is difficult, in that several multi-functional properties are required as input values, which can be taken from the data presented in this study. Moreover, the experimental investigation and proposed models for predicting thermo-mechanical properties, such as Young’s modulus, Poisson’s effect, thermal conductivity, and temperature–frequency-dependent viscoelastic properties, can be used for thermomechanical modeling of epoxy/GO nanocomposites under quasi-static and dynamic loading.

## Figures and Tables

**Table 1:** TGA properties of pristine epoxy and different weight percentages of nanocomposites.

Samples	Weight Loss Temperature (°C)		Heat-Resistance Index (T_HRI_) (°C)	Derivative of TGA Peak Temperature (°C)	
	*T*_5_	*T*_30_		*T*_m1_	*T*_m2_
Epoxy	290.13	343.41	157.83	333.86	574.24
0.05 wt% GO/epoxy	293.46	344.01	158.66	341.33	621.27
0.1 wt% GO/epoxy	297.95	343.38	159.35	335.12	631.66
0.2 wt% GO/epoxy	296.33	344.75	159.44	336.33	617.90

## Data Availability

Not applicable.

## Supplementary Materials

The following are available online at https://www.mdpi.com/article/10.3390/polym13162831/s1, Table S1: Experimental and predicted storage and loss moduli values for pristine epoxy and different GO nanocomposites.
