# Supplementary material for: Experimental Characterization and Modeling Multifunctional Properties of Epoxy/Graphene Oxide Nanocomposites
Source: Polymers (Basel). 2021 Aug 23;13(16):2831. doi: 10.3390/polym13162831 (PMC8401890; doi:10.3390/polym13162831)
Supplement: Supplementary file 1 [file polymers-13-02831-s001.zip › polymers-1330773-supplementary.pdf]

Supporting Information

Experimental Characterization and Modeling Multifunctional Properties of Epoxy/ Graphene Oxide Nanocomposites

K. Naresh[1], K.A. Khan [1*], R. Umer[1]

[1]Department of Aerospace Engineering, Khalifa University of Science and Technology, Abu Dhabi, UAE.

*Corresponding author: kamran.khan@ku.ac.ae. *Tel.: +971-(02) 312 3562*

S1. Thermal properties

S1.1 Storage and loss moduli of epoxy and epoxy/GO nanocomposites

Table S1 shows the comparison between experimental and predicted storage and loss moduli values of pristine epoxy and different weight contents of GO fillers (0.05, 0.1 and 0.2 wt%) added in the epoxy for different frequencies. From the table, the increase in initial or maximum storage modulus and T_g values and the decrease in parameters p and q are observed with the increase in frequency from 0.1 Hz to 30 Hz in all materials. The predicted values are in good agreement with the experimental results.

Table S1 Experimental and predicted storage and loss moduli values for pristine epoxy and different GO nanocomposites.

Material	f (Hz)	Initial storage modulus (GPa) at 23°C		%error	Final storage modulus (GPa) at 150°C		%error	$(E'')_{max}$ (GPa)	$(E'')_{max}$ (GPa)	%error	Parameters	
		Exp.	Pred.		Exp.	Pred.		Exp.	Pred.		p	q
Pristine epoxy	0.1	5.356	5.211	2.782	3.951	3.788	4.303	0.612	0.603	1.493	0.127	2.576
	1	5.475	5.327	2.778	4.020	4.076	1.374	0.637	0.636	0.157	0.121	2.575
	5	5.521	5.361	2.984	4.076	4.061	0.369	0.592	0.590	0.339	0.092	2.150
	10	5.552	5.395	2.910	3.948	3.959	0.278	0.616	0.615	0.163	0.091	2.144
	15	5.569	5.411	2.920	4.192	3.737	12.175	0.659	0.656	0.457	0.090	2.124
	30	5.602	5.475	2.319	3.814	4.309	11.487	0.550	0.547	0.548	0.086	1.948
0.05% G/E	0.1	5.291	5.020	5.398	2.427	2.029	19.616	0.536	0.522	2.682	0.095	3.876
	1	5.376	5.117	5.061	2.283	2.353	2.975	0.546	0.545	0.183	0.094	3.269
	5	5.403	5.136	5.198	2.513	2.239	12.237	0.492	0.489	0.613	0.087	2.265
	10	5.415	5.209	3.955	2.428	2.372	2.361	0.508	0.505	0.594	0.084	2.257
	15	5.421	5.270	2.865	2.399	2.619	8.400	0.507	0.505	0.396	0.083	2.151
	30	5.431	5.271	3.035	2.295	2.415	4.969	0.420	0.420	0	0.08	1.995
0.1% G/E	0.1	4.629	4.498	2.912	1.566	1.481	5.739	0.488	0.485	0.618	0.103	2.895
	1	4.718	4.598	2.609	1.802	1.702	5.875	0.443	0.434	2.074	0.099	2.465
	5	4.741	4.612	2.797	1.766	1.727	2.258	0.429	0.425	0.941	0.098	2.463
	10	4.751	4.622	2.791	1.789	1.766	1.302	0.441	0.434	1.613	0.097	2.461
	15	4.759	4.597	3.524	1.996	1.874	6.510	0.472	0.468	0.855	0.087	2.257
	30	4.77	4.592	3.876	1.871	1.914	2.247	0.469	0.466	0.644	0.084	1.934
0.2% G/E	0.1	8.066	7.877	2.399	4.059	3.664	10.781	0.788	0.780	1.026	0.122	2.976
	1	8.387	8.055	4.122	3.828	3.413	12.159	0.825	0.816	1.103	0.11	2.966
	5	8.389	8.168	2.706	3.836	3.413	12.394	0.827	0.817	1.224	0.109	2.854
	10	8.442	8.147	3.621	3.954	3.437	15.042	0.838	0.828	1.208	0.102	2.745
	15	8.575	8.334	2.892	4.229	3.594	17.668	0.841	0.832	1.082	0.101	2.724
	30	8.647	8.242	4.914	4.113	3.460	18.873	0.782	0.830	5.783	0.091	1.893
